# Performance of Aptima-HPV in the cervical cancer screening program of Portugal: a cost-analysis

**DOI:** 10.1186/s12905-023-02219-0

**Published:** 2023-03-09

**Authors:** Daniel Figueiredo, Inês Ribeiro, Ana Penedones, Diogo Mendes, Carlos Alves, Francisco Batel-Marques, Daniel Pereira da Silva

**Affiliations:** 1 Association for Innovation and Biomedical Research on Light and Image, Coimbra, Portugal; 2 CIDMA – Center for Research and Development in Mathematics and Applications, University of Aveiro, Aveiro, Portugal; 3 Laboratory of Social Pharmacy and Public Health, Faculty of Pharmacy, University of Coimbra, Coimbra, Portugal; 4 SHTAC - Southampton Health Technology Assessment Centre, Faculty of Medicine, University of Southampton, Southampton, UK; 5 CUF Coimbra Hospital, Coimbra, Portugal; 6 Instituto Médico de Coimbra, Coimbra, Portugal

**Keywords:** Early detection of cancer, Papillomavirus infections, Cervical cancer, Costs and cost analysis

## Abstract

**Background:**

Cervical cancer is a major concern to women’s health, being the fourth most common cancer worldwide. A great percentage of these cancer is consequence of an HPV infection, namely from specific genotypes such as 16/18. Portuguese screening program subjects women to a reflex cytology triage every 5 years. Aptima® HPV is a screening test which presents better specificity than other tests which are used in Portugal (Hybrid Capture® 2 and Cobas® 4800) and still have a comparable sensitivity. The present study aims to estimate the number of diagnostic tests and costs that are avoided using Aptima® HPV compared to the use of two other tests, Hybrid Capture® 2 and Cobas® 4800, within the cervical cancer screening programme in Portugal.

**Methods:**

A model, consisting of a decision-tree, was developed to represent the full Portuguese screening program for cervical cancer. This model is used to compare the costs resulting from using Aptima® HPV test versus the other tests used in Portugal, during 2 years. Other outcomes such as the number of additional tests and exams were also computed. This comparison considers the performance of each test (sensitivity and specificity) and assumes an equal price for every test compared.

**Results:**

Cost savings resulting from the use of Aptima® HPV are estimated at approximately €382 million versus Hybrid Capture® 2 and €2.8 million versus Cobas® 4800. Moreover, Aptima® HPV prevents 265,443 and 269,856 additional tests and exams when compared with Hybrid Capture® 2 and Cobas® 4800.

**Conclusions:**

The use of Aptima® HPV resulted in lower costs as well as less additional test and exams. These values result from the greater specificity of Aptima® HPV, which signals less false positive cases and consequently avoids carrying out additional tests.

**Supplementary Information:**

The online version contains supplementary material available at 10.1186/s12905-023-02219-0.

## Background

Cervical cancer is the fourth most common cancer in women worldwide [1]. Approximately 600.000 new cases were diagnosed in 2020 and nearly 340.000 women died because of cervical cancer [1]. In Portugal, the age-standardized incidence and mortality rates were estimated at 10.7 per 100.000 and 3.2 per 100.000, respectively [1].

Screening programs are essential to reduce the incidence of cervical cancer, as well as the associated morbidity and mortality [2]. Cervical cytology is the pioneering method of screening and has contributed to reducing the incidence rate of cervical cancer [3]. However, this screening process needs to be frequently repeated because of its only moderate sensitivity for cervical intraepithelial neoplasia of grade 2 (CIN2) or worse (CIN2+) [4]. Further prevention strategies emerged after human papillomavirus (HPV) infection has been recognized as a major cause of cervical cancer, namely HPV testing and prophylactic vaccination [5].

Data from prospective clinical trials have shown that HPV testing is associated with 60 to 70% higher protection against invasive cervical carcinomas compared to cytology [4]. According to the Portuguese Society of Gynecology clinical consensus, primary HPV testing is the preferred strategy for cervical cancer screening [2]. In addition, the Portuguese legislation foresees HPV testing as the primary screening strategy with 16/18 genotyping to be used as a triage test for immediate referral to colposcopy, while cytology to be used as a triage for other 12 high-risk HPV types [2]. The results of a cost analysis with a hypothetical cohort of ~ 2 million women showed that HPV testing with 16/18 genotyping with reflex cytology triage every 5 years is the most cost-effective cervical cancer screening strategy in Portugal [5]. It improves the rates of detection of cervical cancer and CIN2+ cases, reduces the annual incidence of cervical cancer, as well as the associated mortality, and provides cost-savings when compared to cytology with Atypical Squamous Cells of Undetermined Significance (ASCUS) HPV triage every 3 years [5]. Likewise, the Portuguese Gynecology Society recommends regular cervical cancer screenings, starting at 25 years. It recommends that a cytologic test is offered at this age and these women should be rescreened after 3 years. For women from 30 to 65 years old, the recommendation is to perform an HPV test together with cytology every 5 years [2, 17]. Women with a previous CIN2+ diagnosis should be followed and perform tests more often. No exceptions to this plan are observed for vaccinated women [2, 17].

The Aptima® HPV assay detects E6/E7 viral messenger RNA (mRNA) of 14 high-risk types of HPV (16/18/31/33/35/39/45/51/52/56/58/59/66/68) [6]. The Aptima® HPV 16 18/45 Genotype Assay (Aptima-GT®) detects E6/E7 viral mRNA of 3 high-risk types of HPV (16/18/45) and it is ran on Aptima® HPV assay positive samples to differentiate HPV16 from HPV18/45 (with HPV18 and HPV45 detected together) [7].

The Aptima® test has a similar sensitivity as DNA-based testing, but it has a improved specificity in the detection of HPV positive results and, consequently, CIN2 or CIN2+ cases [8, 9]. The performance of Aptima® in the detection of CIN2+ presents a sensitivity which is comparable to DNA tests, as illustrated by a literature review [10]. In the same review it was possible to observe a higher specificity of Aptima® when compared with other HPV test, both in screening and referral test scenarios [10].

Furthermore, the results of more recently published longitudinal studies indicate that the performance of the Aptima® test for CIN2+ detection is maintained over time as compared to the HC2 test. After 4 years of follow-up, both tests had the same sensitivity (85.0% [95%CI 64.0–94.8]) [11]. In another study, according to the cumulative risks of CIN2+ by the year 6 visit, the relative sensitivity for CIN2+ of the Aptima® test in comparison to the HC2 test was 91.4% [12]. Lastly, in a randomized controlled trial (RCT) which have included participants with baseline HPV negative results, the long-term risk of CIN2+ did no differ between the DNA-based assays and mRNA-based assays after 10 years of follow-up (HR = 0.95 [95%CI 0.79–1.13]) [13].

According to the available evidence, testing with Aptima® originates less false-positive results than DNA-based testing assays. Although sensitivity is a key measure to evaluate the effectiveness of HPV tests, improved specificity is also important to avoid unnecessary use of health resources [14]. The present study aims to estimate the number of diagnostic tests and costs that are avoided by the use of Aptima® HPV compared to the use of Hybrid Capture® 2 and Cobas® 4800 within the cervical cancer screening programme in Portugal. Aptima® HPV is manufactured by Hologic, Hybrid Capture® 2 is manufactured by Qiagen and Cobas® 4800 is manufactured by Roche Diagnostics.

## Methods

To represent the full Portuguese screening program, a decision-tree model was developed. This model consisted of nodes for each step of the screening routine and directed edges which guide the women during this process. Probabilities are assigned to the edges to describe how a generic patient would be guided along the screening process. By considering a cohort composed of Portuguese women aged between 25 and 60, we can estimate the expected number of women who followed each path of the decision tree and quantify the difference in terms of costs and exams performed for each HPV test considered. More details about the model and its design are provided further.

The Portuguese Methodological Guidelines for Economic Evaluation Studies of Health Technologies were followed in this study. These guidelines provide guidance to conduct economic evaluation studies of medicines and medical devices [15]. The National Health Service (NHS) perspective was adopted and all costs and consequences were updated at an annual rate of 4% [15]. A 2-year time horizon was used in the analysis because it is long enough to capture the cost differences between the three cervical cancer screening strategies and it is the maximum length of time that women can be followed after the first HPV diagnostic test. Moreover, this time horizon is coherent with the time horizon recommended by the Portuguese Methodological Guidelines for Economic Evaluation Studies of Health Technologies [15]. Microsoft Excel® 2016 (Microsoft Corporation, Santa Rosa, CA, USA) was used to build the decision tree model and perform calculations.

### Cohort dimension

Table 1 presents the number of women that is expected to be screened for cervical cancer in Portugal. This estimation was based on the number of Portuguese women aged between 25 and 60 years and the proportion of women undergoing cervical cytology in Portugal [16]. No subclass analysis is done regarding age or vaccination and these factors are assumed geographically homogeneous. Thus, 2,199,879 women enter the cohort and are followed during 2 years in the simulation model.

**Table 1:** Number of women enrolled in the cervical cancer screening in Portugal

Geographic region	Women aged 25–60 years old, n =	Cervical cytology (%)	Cohort dimension
Portugal	2,534,423	86.8	2,199,879
Continental Portugal—Norte region	912,749	95.0	867,112
Continental Portugal—Centro region	531,208	89.5	475,431
Continental Portugal—Lisbon region	692,556	81.2	562,355
Continental Portugal—Alentejo region	160,518	75.9	121,833
Continental Portugal—Algarve region	106,498	84.2	89,671
Autonomous region—Azores	63,011	72.5	45,683
Autonomous region—Madeira	67,883	74.4	50,505

References: INE, 2018; Rukhadze et al. [16]

### Screening strategies

The HPV mRNA test Aptima® HPV was compared with two HPV DNA tests (Hybrid Capture® 2 and Cobas® 4800). Hybrid Capture® 2 and Cobas® 4800 were selected as comparators for this study since the first one (Hybrid Capture®) is the standard of care for HPV testing (according to the clinical consensus on cervical cancer screening from the Portuguese Society of Gynecology), and the second one (Cobas® 4800) is the most used screening strategy in Portugal (according to a panel of clinical experts; Additional file 1) [2].

### Decision tree

**Fig. 1:**
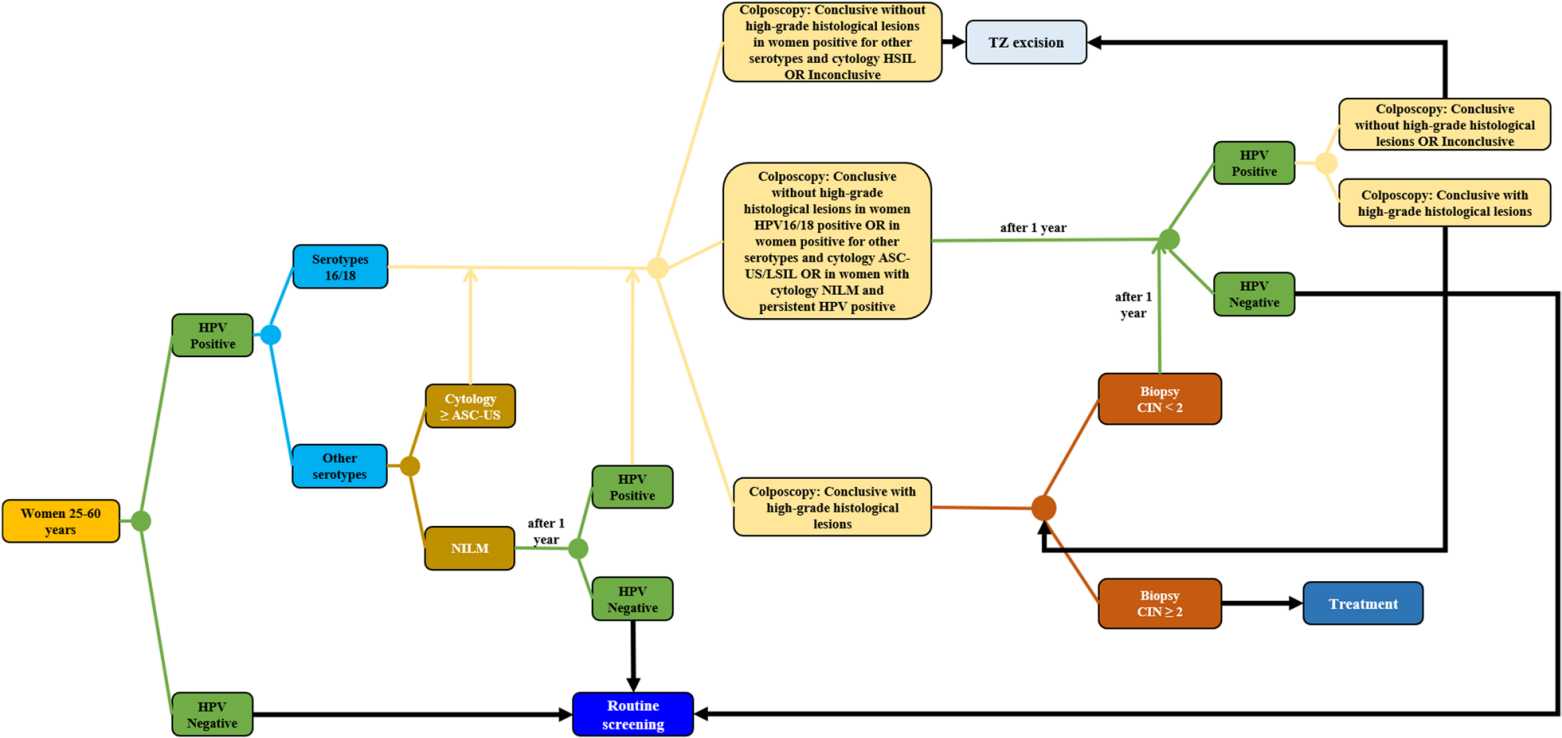
Cervical cancer screening decision tree

The analytical model consists of a decision tree that simulates and compares the screening performance of each HPV testing strategy. The screening algorithm was developed based on the Portuguese legislation, the clinical consensus published by the Portuguese Society of Gynecology on cervical cancer screening, as well as on the opinion of a panel of clinical experts (Fig. 1) [2, 17]. Details on the composition and the methodology of panel of clinical experts are available on the Additional file 1.

The analytical model simulates a cohort of 2,199,879 healthy women aged 25–60 years old. The hypothetical cohort moves between the model states, according to prespecified probabilities in each decision tree node, over a period of 2 years. These probabilities can be applied to estimate the number of women who followed each path within the screening algorithm and estimate the resulting costs and tests used.

According to clinical guidelines, women undergo routine screening for cervical cancer using the HPV test every 5 years. If the result of the HPV test is negative, women undergo routine screening again 5 years later. On the other hand, women testing positive for HPV serotypes 16/18 are referred for colposcopy, while women testing positive for other serotypes of HPV are referred for cytology. Women with a cytology showing ASCUS or worse are referred for colposcopy. Women with a normal result in the cytology are followed-up and re-tested for HPV after 12 months. Women re-testing negative return to routine screening and women re-testing positive are referred for colposcopy. Women with a colposcopy revealing conclusive results without high-grade histological lesions are followed-up and re-tested for HPV after 12 months (HPV-negative women return to routine screening and HPV-positive women undergo new colposcopy). Women with a colposcopy showing conclusive results with high-grade histological lesions undergo biopsy. Women undergo transformation zone (TZ) excision if they have a) inconclusive results in the colposcopy or b) a colposcopy with conclusive results without high-grade histological lesions after cytology High Grade Squamous Intraepithelial Lesion (HSIL). After biopsy results, women with CIN < 2 are followed-up and re-tested for HPV after 12 months (HPV-negative women return to routine screening and HPV-positive women undergo new colposcopy). Women with CIN ≥ 2 initiate treatment. Women with inconclusive results or conclusive results without high-grade histological lesions in the new colposcopy undergo TZ excision. Women with conclusive results with high-grade histological lesions in the new colposcopy repeat biopsy (Fig. 1). Both colposcopy and biopsy are assumed to be 100% sensitive and 100% specific [5].

### Model inputs

#### Performance of screening tests

The transition probabilities within the decision tree were informed by the performance (i.e., sensitivity and specificity) of the HPV tests. The performance of each HPV test was obtained from the study by Cuzick et al. [18], which compared four DNA-based HPV tests (Hybrid Capture 2®, Cobas® 4800, Abbott RealTi*m*e High Risk HPV®, BD HPV®) and two mRNA-based HPV tests (PreTect HPV-Proofer®, Aptima-GT®) [18]. This study included cytology samples from 6000 women who attended screening for a routine 3 or 5 yearly (depending on age) cervical smear [18]. The main outcomes of the study were the specificity and the positive predictive value (PPV) of each HPV test [18]. In addition, inputs from a panel of clinical experts were used to estimate the transition probabilities that could not be found through the literature review (Additional file 1).

Table 2 presents the transition probabilities in each node of the decision tree model. The “probability of a patient testing HPV + ” was estimated based on the sensitivity and specificity of each HPV test (Hybrid Capture® 2, Cobas® 4800 and Aptima® HPV), as well as the prevalence of the HPV infection in Portugal [19]. The prevalence of the HPV infection in women aged between 18 and 64 years old was estimated at 12.7% in the CLEOPATRE study (a cross-sectional population-based study including women who attended gynaecology/obstetrics or sexually transmitted diseases clinics in mainland Portugal) [19].

**Table 2:** Transition probabilities in each node of the decision three model

Node	Transition probabilities (%)	References
*Hybrid Capture® 2*		
Probability (P) of a patient testing HPV+	25.1	Cuzick et al. [18] Pista et al. [19]
P of a patient testing HPV−	74.9	
*Cobas® 4800*		
Probability (P) of a patient testing HPV+	25.9	Cuzick et al. [18] Pista et al. [19]
P of a patient testing HPV−	74.1	
*Aptima® HPV*		
Probability (P) of a patient testing HPV+	20.9	Cuzick et al. [18] Pista et al. [19]
P of a patient testing HPV−	81	
*Hybrid Capture® 2*		
P of an HPV + patient being infected by 16/18 serotypes	22.57	Clinical experts
P of an HPV + patient being infected by other serotypes	77.43	
*Cobas® 4800*		
P of an HPV + patient being infected by 16/18 serotypes	31.33	Cuzick et al. [18]
P of an HPV + patient being infected by other serotypes	68.67	
*Aptima® HPV*		
P of an HPV+ patient being infected by 16/18 serotypes	29.80	Cuzick et al. [18]
P of an HPV+ patient being infected by other serotypes	70.20	
P of an HPV+ patient infected by 16/18 serotypes present a conclusive colposcopy without atypical lesions	66.88	Clinical experts
P of an HPV+ patient infected by 16/18 serotypes present a conclusive colposcopy with atypical lesions	16.72	
P of an HPV+ patient infected by 16/18 serotypes present an inconclusive colposcopy	16.40	
P of an HPV+ patient infected by other serotypes present cytology ≥ ASC-US	78.52	Clinical experts
P of an HPV+ patient infected by other serotypes present cytology NILM	21.48	
P of an HPV+ patient infected by other serotypes and with cytology ASC-US/LSIL present a conclusive colposcopy without atypical lesions	71.91	Clinical experts
P of an HPV+ patient infected by other serotypes and with cytology HSIL present a conclusive colposcopy without atypical lesions	3.79	
P of an HPV+ patient infected by other serotypes and with cytology ≥ ASC-US present a conclusive colposcopy with atypical lesions	7.90	
P of an HPV+ patient infected by other serotypes and with cytology ≥ ASC-US present an inconclusive colposcopy	16.40	
P of an HPV+ patient infected by other serotypes and with cytology NILM present, after a year, a second HPV+ test	10.00	Clinical experts
P of an HPV+ patient infected by other serotypes and with cytology NILM present, after a year, a second HPV- test	90.00	
P of a patient with a second HPV+ test present a conclusive colposcopy without atypical lesions	66.88	Clinical experts
P of a patient with a second HPV+ test present a conclusive colposcopy with atypical lesions	16.72	
P of a patient with a second HPV+ test present an inconclusive colposcopy	16.40	
P of an HPV+ patient infected by 16/18 serotypes with a conclusive colposcopy without atypical lesions present, after a year, a second HPV + test	10.00	Clinical experts
P of an HPV + patient infected by 16/18 serotypes with a conclusive colposcopy without atypical lesions present, after a year, a second HPV− test	90.00	
P of an HPV+ patient infected by other serotypes with cytology ASCUS/LSIL and a conclusive colposcopy without atypical lesions present, after a year, a second HPV+ test	5.00	Clinical experts
P of an HPV + patient infected by other serotypes with cytology ASCUS/LSIL and a conclusive colposcopy without atypical lesions present, after a year, a second HPV− test	95.00	
P of an HPV+ patient infected by other serotypes with cytology NILM and a conclusive colposcopy without atypical lesions present, after a year, a second HPV+ test	5.00	Clinical experts
P of an HPV+ patient infected by other serotypes with cytology NILM and a conclusive colposcopy without atypical lesions present, after a year, a second HPV− test	95.00	
P of a patient with a conclusive colposcopy with atypical lesions present CIN+	63.90	Cuzick et al. [18]
P of a patient with a conclusive colposcopy with atypical lesions present CIN−	36.10	
P of a CIN− patient present, after a year, a second HPV+ test	10.00	Clinical experts
P of a CIN- patient present, after a year, a second HPV− test	90.00	
P of a patient with a second HPV+ test present a second conclusive colposcopy without atypical lesions	71.06	Clinical experts
P of a patient with a second HPV+ test present a second conclusive colposcopy with atypical lesions	12.54	
P of a patient with a second HPV+ test present a second inconclusive colposcopy	16.40	
P of a patient with a second conclusive colposcopy with atypical lesions present CIN+	90.00	Clinical experts
P of a patient with a second conclusive colposcopy with atypical lesions present CIN−	10.00	

References: Cuzick et al. [18]; Pista et al. [19]; Clinical experts (Additional file 1)

#### Costs

The costs (2021 Euros, €) of diagnostic tests (i.e., cytology, colposcopy and biopsy) were considered in the study and were retrieved from Portuguese official sources (Table 3) [20, 21]. The consumption of healthcare resources was estimated based on the Portuguese legislation and the clinical consensus from the Portuguese Society of Gynecology on cervical cancer screening [2, 17]. Unit costs of HPV tests were not considered, since it was assumed that the three HPV tests had the same purchase price. However, the cost of HPV genotyping was considered every time a Hybrid Capture® 2 test was performed because the test alone does not allow to differentiate HPV genotypes (Table 3) [22].

**Table 3:** Use of healthcare resources and associated costs

Healthcare resource	Unit costs	Reference/source
*HPV tests*		
Hybrid Capture® 2	0€	Assumption
Cobas® 4800	0€	Assumption
Aptima® HPV	0€	Assumption
*HPV genotyping after Hybrid Capture® 2*		
HPV genotyping	685.18€*	Qiagen, 2021
*Diagnostic tests*		
Cytology	15.2€	Diário da República, 2018 (Code 30,510)
Colposcopy	14.5€	Diário da República, 2018 (Code 48,180)
Colposcopy + Biopsy	34.4€	Diário da República, 2018 (Code 48,190)

*575£—a formula was used to convert Pounds into Euros [23]

### Model outputs

The comparison between Aptima® HPV versus Hybrid Capture® 2 or Cobas® 4800 was performed considering the number of HPV tests and other diagnostic tests (i.e., cytology, colposcopy, and biopsy) that are avoided with an HPV test versus another. Cost savings resulting from the avoidance of carrying out further HPV tests and other diagnostic tests were estimated to assess the budget impact of using the Aptima® HPV test instead of the other two HPV tests. To compare these tests, it is considered that all women included in the cohort are tested. Since the process is cyclic and may last for a few years, we present the results for the scenarios where each woman is followed: (a) during a 2-year period and (b) until the first colposcopy. Additionally, a scenario where each woman is followed during a single year is presented in the sensitivity analysis.

### Sensitivity analysis

The main source of uncertainty in the analytical model is associated with the transition probabilities; therefore, a deterministic sensitivity analysis (DSA) was used to investigate the sensitivity of the results of the base case scenario after variations of these input parameters. According to the 2019 “Human Papillomavirus and Related Diseases Report” from the ICO/IARC Information Centre on HPV and Cancer, the prevalence of HPV infection among women with normal cervical cytology in Portugal ranges from 10.5 to 25.4% [24]. As such, another DSA was performed to investigate the impact of varying prevalence rates of HPV infection on the results of the study (10.5% and 25.4%). In addition, the unit costs of the diagnostic tests were varied ± 25% (i.e., up and down) to evaluate how they could affect the results. A joint sensitivity analysis was also performed—both the prevalence rates and the costs of the diagnostic tests were varied simultaneously. Finally, the scenarios without discount rate and where the time horizon is 1 year are explored as well.

## Results

### Base case

Screening for cervical cancer with Aptima® HPV avoids carrying out 265,443 and 269,856 additional diagnostic tests (i.e., HPV tests, cytology, colposcopy, and biopsy) after the first HPV test compared to Hybrid Capture® 2 and Cobas® 4800, respectively (Table 4). For the 2 years of this analysis, cost savings resulting from the use of Aptima® HPV are estimated at approximately €382 million versus Hybrid Capture® 2 and €2.8 million versus Cobas® 4800 (Table 4), resulting from savings of 173,45€ and 1,27€ per woman, respectively.

**Table 4:** Additional tests and costs with HC2 and Cobas versus Aptima over 2 years of follow-up

	Diagnostic tests* after the first HPV test, n =	Costs, €	Additional tests* versus Aptima, n =	Additional costs versus Aptima, €
Aptima® HPV	1,137,309	11,794,860 €	–	–
Hybrid Capture® 2	1,399,753	393,354,356.91 €**	262,443	381,559,496.91 €
Cobas® 4800	1,407,165	14,592,550.70 €	269,856	2,797,690.71 €

Legend: Aptima: Aptima® HPV; Cobas: Cobas® 4800; HC2: Hybrid Capture® 2.

*Includes HPV tests and other diagnostic tests (cytology, colposcopy, and biopsy)

**Includes the cost of HPV genotyping after the first and subsequent Hybrid Capture® 2 tests

The number of diagnostic tests that need to be performed by women screened for cervical cancer with Aptima® HPV is lower compared to any other HPV test, irrespectively of the type of diagnostic test (i.e., HPV test, cytology, colposcopy, and biopsy) (Table 5), for the 2 years of analysis.

**Table 5:** Total number of tests with each HPV test and number of additional tests with HC2 and Cobas versus Aptima over 2 years of follow-up

Type of diagnostic test	HPV test			HC2 versus Aptima, n =	Cobas versus Aptima, n =
	Aptima, n =	HC2, n =	Cobas, n =		
HPV test	354,591	430,548	4388	75,957	84,209
Cytology	318,741	428,009	393,923	109,268	75,182
Colposcopy	416,909	489,552	516,127	72,643	99,218
Biopsy	47,068	51,644	58,315	4576	11,247
Total	1,137,309	1,399,753	1,407,165	262,443	269,855

Legend: Aptima: Aptima® HPV; Cobas: Cobas® 4800; HC2: Hybrid Capture® 2.

In addition, screening with Aptima® HPV also avoids carrying out further HPV tests and other diagnostic tests, and therefore generating cost savings, in every Portuguese geographic region individually considered (Table 6).

**Table 6:** Additional tests and costs with HC2 and Cobas versus Aptima over 2 years of follow-up, according to Portuguese geographic regions

Geographic Region	Additional tests, n =	Additional costs, €
*Continental Portugal—Norte region*		
Hybrid Capture® 2 versus Aptima® HPV	103,446	150,396,736.42 €
Cobas® 4800 versus Aptima® HPV	106,367	1,102,746.90 €
*Continental Portugal—Centro region*		
Hybrid Capture® 2 versus Aptima® HPV	56,718	82,461,472.06 €
Cobas® 4800 versus Aptima® HPV	58,320	604,628.36 €
*Continental Portugal—Lisbon region*		
Hybrid Capture® 2 versus Aptima® HPV	67,088	97,538,116.86 €
Cobas® 4800 versus Aptima® HPV	68,983	715,174.14 €
*Continental Portugal—Alentejo region*		
Hybrid Capture® 2 versus Aptima® HPV	14,535	21,131,433.38 €
Cobas® 4800 versus Aptima® HPV	14,945	154,941.01 €
*Continental Portugal—Algarve region*		
Hybrid Capture® 2 versus Aptima® HPV	10,698	15,553,100.72 €
Cobas® 4800 versus Aptima® HPV	11,000	114,039.27 €
*Autonomous region—Azores*		
Hybrid Capture® 2 versus Aptima® HPV	5450	7,923,513.82 €
Cobas® 4800 versus Aptima® HPV	5604	58,097.21 €
*Autonomous region—Madeira*		
Hybrid Capture® 2 versus Aptima® HPV	6025	8,759,864.81 €
Cobas® 4800 versus Aptima® HPV	6195	64,229.54 €

Legend: Aptima: Aptima® HPV; Cobas: Cobas® 4800; HC2: Hybrid Capture® 2.

### Results until the first colposcopy

When the analysis was conducted considering only the diagnostic procedures carried out until the first colposcopy, the Aptima® HPV test still avoided diagnostic tests and saved costs compared to Hybrid Capture® 2 and Cobas® 4800 (Table 7).

**Table 7:** Additional tests and costs with HC2 and Cobas versus Aptima until the first colposcopy

	Diagnostic tests* after the first HPV test, n =	Costs, €	Additional tests* versus Aptima, n =	Additional costs versus Aptima, €
Aptima® HPV	786,195	10,626,532.78 €	–	−
Hybrid Capture® 2	989,993	392,078,694.14 €**	203,798	381,452,161.36 €
Cobas® 4800	972,460	13,144,973.31 €	186,265	2,518,440.53 €

Legend: Aptima: Aptima® HPV; Cobas: Cobas® 4800; HC2: Hybrid Capture® 2.

*Includes HPV tests and other diagnostic tests (cytology, colposcopy, and biopsy)

**Includes the cost of HPV genotyping after the first and subsequent Hybrid Capture® 2 tests

### Sensitivity analysis

The sensitivity analysis showed that the use of Aptima® HPV is also associated with fewer tests and lower costs compared to the use of Hybrid Capture® 2 and Cobas® 4800 when both the prevalence rates of HPV infection (Table 8) and the costs of the diagnostic tests (cytology, colposcopy, and biopsy; Table 9) were varied. The results also remained favourable to Aptima® HPV versus both comparators when both parameters were changed simultaneously (Table 10). The scenarios where the time horizon is set to 1 year (Table 11) and the one with no discount rate (Table 12) maintain the same tendency and the results for Aptima® HPV, Hybrid Capture® 2 and Cobas® 4800 are affect in a smaller scale.

**Table 8:** Sensitivity analysis results for a time horizon of 2 years: variation of HPV prevalence

Prevalence	Additional tests* versus Aptima, n =	Additional costs versus Aptima, €
*Lower value (10.5%)*		
Hybrid Capture® 2	265,652	354,096,906.62 €**
Cobas® 4800	276,698	2,868,712.91 €
*Higher value (25.4%)*		
Hybrid Capture® 2	243,924	540,093,540.88 €**
Cobas® 4800	230,360	2,387,698.92 €

Legend: Aptima: Aptima® HPV.

*Includes HPV tests and other diagnostic tests (cytology, colposcopy, and biopsy)

**Includes the cost of HPV genotyping after the first and subsequent Hybrid Capture® 2 tests

**Table 9:** Sensitivity analysis results for a time horizon of 2 years: variation of costs

Costs of diagnostic tests*	Additional tests** versus Aptima, n =	Additional costs versus Aptima, €
*Lower value (−25%)*		
Hybrid Capture® 2	262,443	305,247,597.53 €
Cobas® 4800	269,856	2,238,152.57 €
*Higher value (+ 25%)*		
Hybrid Capture® 2	262,443	476,949,371.14 €
Cobas® 4800	269,856	3,497,113.38 €

*Includes cytology, colposcopy, and biopsy

**Includes HPV tests and other diagnostic tests (cytology, colposcopy, and biopsy)

Includes the cost of HPV genotyping after the first and subsequent Hybrid Capture® 2 tests

**Table 10:** Sensitivity analysis results for a time horizon of 2 years: variation of prevalence and costs

	Additional tests** versus Aptima, n =	Additional costs versus Aptima, €
Lower values for costs* (− 25%) and prevalence (10.5%)		
Hybrid Capture® 2	265,652	283,277,525.29 €
Cobas® 4800	276,698	2,294,970.33 €
Higher values for costs* (+ 25%) and lower value for prevalence (10.5%)		
Hybrid Capture® 2	265,652	442,621,133.27 €
Cobas® 4800	276,698	3,585,891.13 €
Lower values for costs* (− 25%) and higher value for prevalence (25.4%)		
Hybrid Capture® 2	243,924	432,074,832.70 €
Cobas® 4800	230,360	1,910,159.13 €
Higher values for costs* (+ 25%) and prevalence (25.4%)		
Hybrid Capture® 2	243,924	675,116,926.10 €
Cobas® 4800	230,360	2,984,623.65 €

*Includes cytology, colposcopy, and biopsy

**Includes HPV tests and other diagnostic tests (cytology, colposcopy, and biopsy)

Includes the cost of HPV genotyping after the first and subsequent Hybrid Capture® 2 tests

**Table 11:** Sensitivity analysis results for a time horizon of 1 year

	Diagnostic tests* after the first HPV test, n =	Costs, €	Additional tests* versus Aptima, n =	Additional costs versus Aptima, €
Aptima® HPV	782,718	10,531,090.77 €	–	–
Hybrid Capture® 2	969,205	391,950,533.69 €**	186,486	381,419,442.92 €
Cobas® 4800	968,365	13,027,190.26 €	185,647	2,495,928.49 €

*Includes cytology, colposcopy, and biopsy

**Includes HPV tests and other diagnostic tests (cytology, colposcopy, and biopsy)

Includes the cost of HPV genotyping after the first and subsequent Hybrid Capture® 2 tests

**Table 12:** Sensitivity analysis results for a time horizon of 2 year and no discount rate

	Costs, €	Additional costs versus Aptima, €
Aptima® HPV	11,809,879.39 €	–
Hybrid Capture® 2	393,371,446.36 €*	381,561,664.97 €
Cobas® 4800	14,611,150.11 €	2,891,270.72 €

*Includes HPV tests and other diagnostic tests (cytology, colposcopy, and biopsy)

Includes the cost of HPV genotyping after the first and subsequent Hybrid Capture® 2 tests

#### Prevalence

The number of tests and costs avoided by Aptima® HPV compared to Hybrid Capture® 2 and Cobas® 4800 have not significantly changed when the lower rate of prevalence (10.5%) of HPV infection was assumed. However, the results have significantly changed when the higher value of prevalence (25.4%) was used (Table 8). Aptima® HPV has remained as the best option (i.e., less costly) in both scenarios.

#### Costs

Table 9 describes the number of additional tests and costs associated with the use of Hybrid Capture® 2 and Cobas® 4800 compared to the use of Aptima® HPV when the costs of the diagnostic tests (cytology, colposcopy, and biopsy) were increased and decreased by 25%. In both scenarios, Aptima® HPV is cost saving compared to the other two HPV tests.

#### Joint sensitivity analysis: prevalence and costs

Table 10 describes the results of the analysis when both the prevalence of HPV infection in Portugal and the costs of the diagnostic tests (cytology, colposcopy, and biopsy) were varied simultaneously. Aptima ® HPV remained as the most cost saving option, irrespectively of the variation.

#### Time horizon

Table 11 presents the results relative a time horizon of 1 year. Aptima is the most cost saving option, but the dimension of the savings is slightly lower.

#### Discount rate

Finally, a scenario with no discount rate for costs is explored and the corresponding results are presented in Table 12, where very small relative differences are observed.

## Discussion

This study compared the number of diagnostic tests and costs associated with the use of Aptima® HPV (Hologic) compared to those associated with the use of Hybrid Capture® 2 (Qiagen) and Cobas® 4800 (Roche Diagnosis) within the cervical cancer screening programme in Portugal. Based on the available evidence, Aptima® HPV avoids additional HPV tests and other diagnostic tests (i.e., cytology, colposcopy, and biopsy), therefore generating significant cost savings when compared to the other two HPV tests considered in this analysis. The cervical cancer screening strategy is homogeneous throughout all national territory, leading to cost savings in all regions. By assuming that the purchase prices of the three HPV tests are equal, the cost savings associated with the use of Aptima® HPV results essentially from its improved specificity compared to the other two HPV tests. In addition to that, the need of HPV genotyping upon the use of the Hybrid Capture® 2 test contributes to further increase the amount of costs saved with Aptima® HPV versus the former.

This analysis is in line with the methodology of a previous study that compared the cost-effectiveness of high-risk HPV testing using a DNA-based assay and a mRNA-based assay under the US cervical cancer screening guidelines [8]. The same methodology was also used in recent studies in Europe, namely in the United Kingdom [25] and in Spain [26], where it was concluded that testing for HPV infection with mRNA-based assays generate less costs and consumes less health resources (i.e., number of colposcopies and additional tests) than testing with DNA-based assays.

According to the results of this 2-year time horizon analysis, the number of diagnostic tests avoided by Aptima® HPV versus Hybrid Capture® 2 (n = 262,444) was lower than the number resulting from the comparison between Aptima® HPV and Cobas® 4800 (n = 269,856). However, the costs difference between Aptima® HPV and Hybrid Capture® 2 was greater. The reason is that Hybrid Capture® 2 test does not allow to differentiate HPV genotypes and therefore the cost of genotyping needed to be added to Hybrid Capture® 2, making it the most expensive option. The number of cytology procedures avoided by Aptima® HPV versus Hybrid Capture® 2 was greater than versus Cobas® 4800. This difference is a consequence of the performance of each HPV test—sensitivity and specificity. Hybrid Capture® 2 is associated with an increased probability of a patient with an HPV+ test being infected by other serotypes compared to Aptima® HPV and Cobas® 4800. On the contrary, Cobas® 4800 is associated with an increased probability of a patient with an HPV+ test being infected by 16/18 serotypes, leading to the need to perform more colposcopies and biopsies to confirm the diagnosis.

According to the decision tree model, the proportion of women who are submitted to HPV re-test after the first colposcopy is low. Only women for whom the first colposcopy had conclusive results without high-grade histological, or those with CIN < 2 are re-tested for HPV in a follow-up after 12 months. Moreover, both colposcopy and biopsy were assumed to be 100% sensitive and 100% specific. Therefore, both the number of tests avoided, and the costs saved by Aptima® HPV compared to the other two HPV tests have not significantly changed when the analysis was carried out only until the first colposcopy.

The sensitivity analysis showed that the conclusions obtained in the base case scenario were robust, since the direction of the results remained unchanged in every alternative scenario. Additionally, the time horizon and the discount rate were shown to impact the results in a lower scale than the costs and prevalence of HPV. Noteworthy, the variation of the prevalence rate of HPV infection significantly affects the results of this study. Given that the sensitivity of Aptima® HPV is comparable to the one of Cobas® 4800 and Hybrid Capture® 2 (97.5% for both), the consequences of varying the prevalence rate of HPV infection are mainly explained by the specificity of each test (90.2%, 84.5 and 85.4%, respectively). Since mRNA-based tests, such as Aptima® HPV, have greater specificity than DNA-based tests, the advantage of the formers are more noticeable when the prevalence of HPV infection in the population is lower. When the prevalence of HPV infection increases, the total number of false positives decreases (i.e., there is fewer negative cases in the population that may be incorrectly classified as positives by the HPV test). Thus, if there is a lower number of false positives, the number of subsequent diagnostic tests is also going to be lower. That is why the number of subsequent diagnostic tests avoided by Aptima® HPV versus Cobas® 4800 was lower in the sensitivity analysis with a higher prevalence rate of HPV infection (i.e., in a scenario with less cases of false positives in the population) compared to the base case scenario analysis. The same rationale is applicable for the comparison between Aptima ® HPV and Hybrid Capture® 2.

Considering the analysis of costs, the savings of Aptima ® HPV versus Cobas® 4800 are higher when the prevalence is lower due to the higher number of diagnostic tests avoided (as explained before). Regarding the comparison of Aptima® HPV versus Hybrid Capture® 2, an increase of the prevalence rate of HPV infection in Portugal leads to an increase in the total number of HPV genotyping tests. Because of this, the use of Aptima® HPV in this scenario generates greater cost savings versus Hybrid Capture® 2. Conversely, when the prevalence of HPV infection decreases the number of HPV genotyping tests avoided and cost savings decrease.

The results of the present study have not significantly changed when the costs of the additional diagnostic tests (colposcopies, biopsies, and cytology tests) were varied, with little impact in the magnitude of the costs avoided by using Aptima® HPV.

There are some limitations that should be pointed out. First, this cost analysis was based on an analytical model which simulates the Portuguese cervical cancer screening program. Although few steps of the screening program were obtained from literature, a panel of clinical experts was needed to describe the procedures used to follow-up women after their first HPV test. In addition, many transition probabilities were informed by the experts, since these data were not available in the scientific literature. Therefore, there are some uncertainties intrinsically associated with this decision tree model.

Second, the performance characteristics of the HPV tests were retrieved from a study conducted in the United Kingdom, in which six HPV diagnostic tests have been compared, including Aptima® HPV, Cobas® 4800 and Hybrid Capture® 2 [18]. Studies comparing HPV diagnostic tests in a screening Portuguese setting were not found in the scientific literature. Nevertheless, there is no evidence that differences in populations may have a significant effect in the performance of the HPV tests.

Third, the prevalence of HPV infection among women with normal cytology varies considerably. In Europe, the rates of HPV infection prevalence range from 2.0 to 48.3% [24]. The same occurs with HPV types 16/18, which prevalence in Europe varies between 1.5 and 18.0% [24]. In Portugal, the prevalence rates of HPV infection among women range from 10.5 to 25.4% [24]. Yet, although the sensitivity analysis demonstrated that the results of this study vary significantly as the prevalence rate of HPV infection changes, Aptima® HPV remained as the most cost saving option when compared with both Cobas® 4800 and Hybrid Capture® 2 in every scenario.

Fourth, in line with a similar study, it was assumed that patients would perfectly adhere to the screening programme, diagnosis and follow-up recommendations [8]. However, deviations may occur, since some women may not comply with follow-up times or screening intervals.

Fifth, only two HPV DNA-based tests were compared with Aptima® HPV. According to the consensus of the Portuguese Society of Gynecology, Hybrid Capture® 2 test is the standard of care on cervical cancer screening. The panel of the experts in gynecology which provided inputs for this study elected Cobas® 4800 as the most used screening strategy in Portugal. There are additional HPV tests available on the market which were not considered in this analysis, because they are rarely used. Nonetheless, the results of this study may need to be updated if the HPV tests considered as the standard of care in cervical cancer screening changes in the future.

Sixth, we assume equal prices for the three tests compared. Although this is a standard practice in cost analysis, one must bear in mind this assumption when interpretating these results.

## Conclusions

In conclusion, the results of this study have shown that the use of Aptima® HPV is cost saving compared to the use of both Hybrid Capture® 2 and Cobas® 4800, supporting the replacement of the DNA HPV tests currently in use within the cervical cancer screening programme in Portugal by Aptima® HPV.

### Supplementary Information

**Additional file 1**. Supplemental material.

## Data Availability

All data generated or analysed during this study are included in this published article and its Additional file 1.

## Abbreviations

ASCUS: Atypical Squamous Cells of Undetermined Significance
CIN: Cervical intraepithelial neoplasia
CIN2: Cervical intraepithelial neoplasia of grade 2
CIN2+: Cervical intraepithelial neoplasia of grade 2 or worse
DSA: Deterministic sensitivity analysis
DNA: Deoxyribonucleic acid
HSIL: High Grade Squamous Intraepithelial Lesion
HPV: Human papillomavirus
IARC: International Agency for Research on Cancer
ICO: Catalan Institute of Oncology
mRNA: *messenger* Ribonucleic acid
NHS: National Health Service
P: Probability
TZ: Transformation zone
