# Correction: Performance of Aptima-HPV in the cervical cancer screening program of Portugal: a cost-analysis

**DOI:** 10.1186/s12905-024-02927-1

**Published:** 2024-02-08

**Authors:** Daniel Figueiredo, Inês Ribeiro, Ana Penedones, Diogo Mendes, Carlos Alves, Francisco Batel-Marques, Daniel Pereira da Silva

**Affiliations:** 1 Association for Innovation and Biomedical Research on Light and Image, Coimbra, Portugal; 2 CIDMA - Center for Research and Development in Mathematics and Applications, University of Aveiro, Aveiro, Portugal; 3 Laboratory of Social Pharmacy and Public Health, Faculty of Pharmacy, University of Coimbra, Coimbra, Portugal; 4 SHTAC - Southampton Health Technology Assessment Centre, Faculty of Medicine, University of Southampton, Southampton, UK; 5 CUF Coimbra Hospital, Coimbra, Portugal; 6 Instituto Médico de Coimbra, Coimbra, Portugal

**BMC Women’s Health (2023) 23:96**

10.1186/s12905-023-02219-0

Following publication of the original article [1], the text need to be added in the acknowledgement section “Óscar Lourenço, whose affiliation is Univ. Coimbra, CeBER, Faculty of Economics, Av. Dias da Silva 165, 3004 − 512 Coimbra, has participated in the elaboration of this paper and should be considered as an author. He was not included in the author list due to an unnoticed error”. So, the acknowledgement section will read as “The authors would like to acknowledge to the expert panel members who kindly accepted to participate in this work: Dra. Olga Ilheu, Dra. Amélia Pedro, Dr. José Moutinho, Dra. Fernanda Loureiro, Dra. Paula Borralho, and Dra. Ana Quintas. D. Figueiredo acknowledges the support of CIDMA through the Portuguese Foundation for Science and Technology (UIDB/04106/2020). Óscar Lourenço, whose affiliation is Univ. Coimbra, CeBER, Faculty of Economics, Av. Dias da Silva 165, 3004 − 512 Coimbra, has participated in the elaboration of this paper and should be considered as an author. He was not included in the author list due to an unnoticed error.”

The original article has been corrected.
