# Supplementary material for: Performance of Aptima-HPV in the cervical cancer screening program of Portugal: a cost-analysis
Source: BMC Womens Health. 2023 Mar 9;23:96. doi: 10.1186/s12905-023-02219-0 (PMC9999620; doi:10.1186/s12905-023-02219-0)
Supplement: Supplementary file 1 — Additional file 1. Supplemental material. [file 12905_2023_2219_MOESM1_ESM.docx]

# Supplemental Material

## Expert panel

After a literature search in order to identify studies that provide evidence matching the requirements of this analysis, such as data associated to the screening and diagnosis of cervical cancer in Portugal, it was not possible to find studies that met these needs.

Thus, in the absence of literature information with the essential evidence, the team responsible for the development of this cost analysis decided to use an expert panel of Portuguese physicians specialized in the management of cervical cancer. Although this method was not preferential, it has been successful used in the development of pharmacoeconomic studies. Additionally, the “Portuguese Methodological Guidelines for Economic Evaluation Studies of Health Technologies” [Perelman et al, 2019] recognizes this methodology as an acceptable method to obtain information. It recommends however that the composition of the expert panel was totally described and justified in terms of representativeness of the participants.

The composition of the expert panel was idealized to fulfill two criteria of representativeness: territorial representativeness and clinical representativeness. Concerning territorial representativeness, it was selected physicians that represent the principal health centers in the North, Center and South of the country. The clinical representativeness criterion requires experts with experience in the management of several variants of the disease. The management of cervical cancer is performed by gynecologists, anatomical pathologists, and specialists in public health.

In line with the two criteria described above, Table A presents physicians that were included in the expert panel.

Table A: Expert panel

| Name | Medical specialty | Work place |
| --- | --- | --- |
| Daniel Pereira da Silva | Gynecology | Instituto Português de Oncologia, Coimbra |
| Ana Quintas | Gynecology | Hospital Garcia de Orta, Almada |
| José Moutinho | Gynecology | Instituto Português de Oncologia, Porto |
| Amélia Pedro | Gynecology | Hospital Fernando Fonseca, Amadora |
| Paula Borralho | Anatomical pathology | Faculdade de Medicina da Universidade de Lisboa |
| Olga Ilhéu | Anatomical pathology | Instituto Português de Oncologia, Coimbra |
| Fernanda Loureiro | Public Health | Administração Regional de Saúde do Centro, Coimbra |

It was considered that the seven physicians that comprise the panel meet the representativeness criteria, both territorial and clinical. Thus, their estimates represent a good approximation that reflects precisely the conditions associated to the management of cervical cancer in Portugal.

To gather the estimates from the experts, each one answered a questionnaire with the relevant information. The questions aimed to identify the average medical practice for each expert. The questionnaire focused on the following areas:

- Validation of the Portuguese cervical cancer screening algorithm;
- HPV tests most used in Portugal (comparators)
- Probability transitions estimates.

All collected information was then synthetized (through descriptive statistics), leading to the final set of responses, imputed to the model.

**REFERENCES**

Perelman J, Soares M, Mateus C, Duarte A, Faria R, Ferreira L, Saramago P, Veiga P, Furtado C, Caldeira S, Teixeira MC, Sculpher M (2019): Orientações Metodológicas para Estudos de Avaliação Económica. INFARMED - Autoridade Nacional do Medicamento e Produtos de Saúde, I.P., Lisboa.

Disponível online em [www.infarmed.pt](http://www.infarmed.pt)
